# Engineered Soluble Monomeric IgG1 Fc with Significantly Decreased Non-Specific Binding

**DOI:** 10.3389/fimmu.2017.01545

**Published:** 2017-11-13

**Authors:** Chunyu Wang, Yanling Wu, Lili Wang, Binbin Hong, Yujia Jin, Dan Hu, Gang Chen, Yu Kong, Ailing Huang, Guoqiang Hua, Tianlei Ying

**Affiliations:** ^1^Key Laboratory of Medical Molecular Virology of Ministries of Education and Health, School of Basic Medical Sciences, Fudan University, Shanghai, China; ^2^Institute of Radiation Medicine, Fudan University, Shanghai, China

**Keywords:** monomeric IgG1 Fc, non-specific binding, neonatal Fc receptor, half-life, monoclonal antibody

## Abstract

Due to the long serum half-life provided by the neonatal Fc receptor (FcRn) recycling, the IgG1 Fc has been pursued as the fusion partner to develop therapeutic Fc-fusion proteins, or as the antibody-derived scaffold that could be engineered with antigen-binding capabilities. In previous studies, we engineered the monomeric Fc by mutating critical residues located on the IgG1 Fc dimerization interface. Comparing with the wild-type dimeric Fc, monomeric Fc might possess substantial advantages conferred by its smaller size, but also suffers the disadvantage of non-specific binding to some unrelated antigens, raising considerable concerns over its potential clinical development. Here, we describe a phage display-based strategy to examine the effects of multiple mutations of IgG1 monomeric Fc and, simultaneously, to identify new Fc monomers with desired properties. Consequently, we identified a novel monomeric Fc that displayed significantly decreased non-specificity. In addition, it exhibited higher thermal stability and comparable pH-dependent FcRn binding to the previous reported monomeric Fc. These results provide baseline to understand the mechanism underlying the generation of soluble IgG1 Fc monomers and warrant the further clinical development of monomeric Fc-based fusion proteins as well as antigen binders.

## Introduction

Monoclonal antibodies (mAbs) have become the fastest growing class of biological therapeutics, with over 50 such molecules approved by the FDA for therapeutic use and hundreds more currently in clinical development (1–5). Most of the mAbs on the market are IgG1 antibodies partially because of the long *in vivo* half-life conferred by their pH-dependent association with the neonatal Fc receptor (FcRn). The Fc region of IgG1 can bind to FcRn in the acidic environment of the endosome after antibody internalization, protecting antibody from degradation until its back to the cell surface for re-release into circulation at neutral pH (6, 7). This mechanism enables a less frequent dosing and/or lower dose than other biologics lacking the IgG1 Fc region (8–10). Therefore, it is promising to develop novel long-acting protein therapeutics based on IgG1 Fc by fusing Fc to otherwise fast-turnover therapeutic proteins. IgG1 Fc is also being pursued as the novel antibody-derived scaffold that could be engineered with antigen-binding capabilities, aiming to overcome the limitations of full-size mAbs, such as poor tissue penetration, high manufacturing cost, and hindered access to sterically restricted epitopes (11–14).

However, until now, only a few Fc-fusion proteins have been approved by FDA, and even fewer Fc-based antigen binders are in pre-clinical and clinical development. The applicability of IgG1 Fc is largely hampered by its homodimeric nature, resulting in the large size of fusion proteins and the inability to generate a monovalent fusion construct. Besides, wild-type IgG1 Fc contains multiple binding sites in order to interact with a variety of distinct cell receptors and complement proteins, e.g., FcRn, Fcγ receptors (FcγRs), complement component 1q (C1q), tripartite motif-containing protein 21 (TRIM21) (15–18), and the introduction of mutant sites in the Fc region are prone to induce non-specific binding to irrelevant proteins. Therefore, a key challenge is to avoid the introduction of non-specific cross-reactivity during the engineering of IgG1 Fc for desired properties (19), e.g., reduced molecular weight, improved stability and solubility, or enhanced *in vivo* half-life and effector functions.

**Figure 1:**
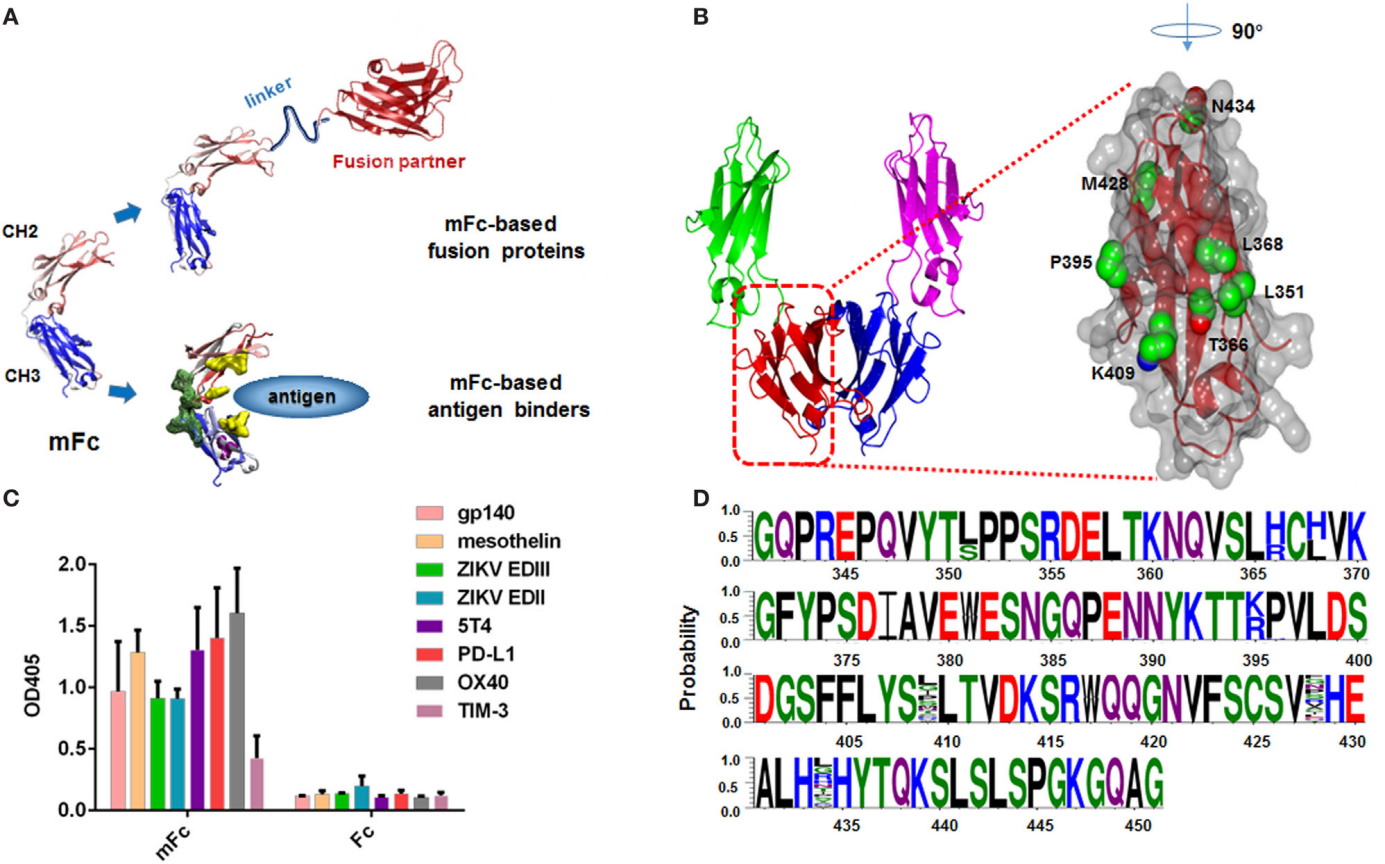
Design and construction of a large Fc mutant library. **(A)** Representative of mFc used to generate mFc-based fusion proteins or mFc-based antigen binders. **(B)** Structure of human IgG1 Fc CH3 domain, showing key residues in its dimerization interface and neonatal Fc receptor-binding region (Protein Data Bank code 2WAH). **(C)** Binding of 2µM mFc and Fc to eight recombinant viral proteins and cancer-related antigens (HIV-1 gp140, ZIKV EDII, ZIKV EDIII, mesothelin, 5T4, PD-L1, OX40, TIM-3) measured by ELISA. The recombinant antigens were coated on ELISA plates, and HRP conjugated anti-FLAG antibody was used for detection of Fc and mFc. **(D)** Sequences of 30 randomly selected clones from Fc mutant library, showing its diversity.

In the previous studies, we generated a large phage library (~1.3 × 10^9^ diversity) of IgG1 Fc molecules with extensive mutations in the dimerization interface, and developed a phage display-based multiple panning/screening strategy to search for monomeric Fc constructs (20, 21). One of the identified Fc monomer, designated as mFc, has only four mutations to the wild-type IgG1 Fc, and is half the size (molecular mass 27 vs. 54 kDa). It was used to generate mFc-based fusion proteins due to the comparable pharmacokinetics to dimeric Fc. Recently, we also used mFc as the scaffold for generating antigen binders (Figure 1A). By introducing point mutations in the CH2 domain and CDR3-grafting onto the CH3 domain of the mFc scaffold, we successfully identified panels of high-affinity mFc-based binders against viral and cancer antigens (unpublished data). However, we found that these binders and mFc *per se* exhibited different extents of non-specific binding at high protein concentrations (>1 μM) to some unrelated antigens, raising considerable concerns over their potential clinical development. We hypothesized that the non-specificity came from the introduced mutations at the IgG1 Fc dimerization interface. Hence, a current priority is to understand the role each of the four mFc mutations plays in the formation of monomer and introduction of non-specificity, and translate this information into the design of a new generation of monomeric IgG1 Fc constructs with minimal non-specific bindings.

Here, we describe a novel strategy to examine the effects of multiple mutations of IgG1 mFc and, simultaneously, to identify new Fc monomers with desired properties. Based on our knowledge of Fc homo-dimerization, we designed a phage display library that enables a combination of rational and random scanning mutagenesis of seven mFc residues (positions Leu-351, Thr-366, Leu-368, Pro-395, Lys-409, Met-428, and Asn-434). All these residues have been previously identified to impact the Fc dimerization or FcRn binding (Figure 1B) (6, 15, 20–22). The displayed variants were then subjected to five rounds of extensive bio-panning based on their conformation, specific and non-specific FcRn binding. While the non-monomeric mutants have diverse sequences, the identified IgG1 Fc monomers have similar positive-charged amino acids at positions 366 and 368 (T366R, L368H), suggesting a critical role of these mutations in the disruption of Fc dimerization. Importantly, we identified a novel monomeric Fc mutant that still maintained FcRn-binding capability, and exhibited significantly decreased non-specific binding as compared to the previously developed monomeric IgG1 Fc mutants. These results provide baseline to understand the mechanism underlying the generation of soluble IgG1 Fc monomers and warrant the further clinical development of mFc-based fusion proteins as well as antigen binders.

## Materials and Methods

### Library Construction and Selection of Fc Mutant Clones

A large phage display library, with an estimated diversity of 1.28 × 10^5^, was constructed by site-directed mutation of four residues of human IgG1 Fc (Leu-351, Thr-366, Leu-368, and Pro-395), and randomly mutation of the other three residues, one located at the CH3 dimer interface (Lys-409) and the other two located within the FcRn-binding site of CH3 (Met-428 and Asn-434). The synthesized gene (Genewiz Inc., Suzhou, China) was cloned into the pComb3x phagemid as described previously (20, 21). Amplified libraries with 10^12^ phage-displayed Fc mutants were used in the following bio-panning. After two rounds of protein G panning using protein G magnetic beads (Roche Applied Science), the phages capable of binding to protein G were amplified from the TG1 cells and used in the bio-panning against FcRn. The FcRn, being composed of alpha chain (or heavy chain) and a beta 2 microglobulin noncovalently linked at the 1:1 M ratio, was expressed in mammalian cells and purified as a soluble protein as previously described. Next, 10^12^ phages were mixed with phosphate-buffered saline (PBS, pH 6.0) and incubated with biotin-labeled FcRn conjugated to magnetic beads (Invitrogen, Carlsbad, CA, USA) for 1.5 h at room temperature. After incubation, the beads were washed two times for the first round and increase progressively for the later rounds with PBS (pH 6.0) containing 0.05% Tween 20. The bound phages were eluted with PBS (pH 7.4), amplified by infecting TG1 cells along with helper phage M13KO7 (Thermo Fisher). Positive clones that bound to FcRn at pH 6.0, but did not bind at pH 7.4 were randomly picked from the fifth selection round bio-panning using monoclonal phage ELISA.

### Expression and Purification of Fc Mutants

The selected clones were sequenced, and plasmids extracted from these clones were used for transformation of HB2151 cells. A single and freshly transformed colony was added to 3 ml 2YT medium with 100 µg/ml ampicillin and 2% (w/v) glucose, incubated at 37°C with vigorous shaking at 250 rpm for 3–4 h, and then transferred into 200 ml of SB medium with 100 µg/ml ampicillin for continued incubation until optical density of the culture at 600 nm reached 0.6. Next, IPTG (isopropyl-1-thio-β-d-galactopyranoside) was added to a final concentration of 1 mM to induce protein expression, and the culture was further incubated overnight at 30°C, 250 rpm. Bacteria were collected by centrifugation at 8,000 rpm for 10 min and re-suspended in 30 ml PBS buffer. Polymixin B (Sigma-Aldrich) (0.5 µm/ml) was added to the bacteria solution (1:1,000). After 30 min incubation with rotation at 250 rpm at 30°C, the suspension was centrifuged at 8,000 rpm for 10 min at 4°C. Proteins were purified using protein G column (Roche Applied Science) according to the manufacturer’s protocol. The degree of protein purity was determined by SDS-PAGE, and protein concentration was measured spectrophotometrically.

### Size Exclusion Chromatography (SEC)

Protein samples were prepared at concentrations of 400–500 µg/ml in PBS buffer. Each sample (250 µg) was injected onto an analytical Superdex 75 10/300 GL column (GE Healthcare) connected to an FPLC ÄKTA BASIC pH/C system (GE Healthcare). PBS was used as the running buffer at the flow rate 0.5 ml/min, and the eluted proteins were monitored at 280 nm. Fc (50 kDa) and mFc (25 kDa) were used to define the molecular mass. A minimum of three independent experiments was performed.

### Circular Dichroism (CD)

The CD spectra were collected on a Jasco J-815 spectropolarimeter (Jasco International). The protein samples were dissolved in PBS at a final concentration of 0.25 mg/ml. For evaluation of thermal stability, the spectra were analyzed by monitoring the molar ellipticity changes at 216 nm as a function of temperature increase (0.1 cm path length). The instrument was programmed to acquire spectra at 1°C intervals over the range 20–100°C. A minimum of three independent experiments was performed.

### Binding ELISA

ELISA was used to determine the binding capability of the selected proteins to various unrelated antigens. All Fc mutants (mFc, 1-B10-9, 1-D1-15) were expressed in *E. coli* HB2151, as described above. Antigens (gp140, mesothelin, ZIKV EDII, ZIKV EDIII, 5T4, PD-L1, OX40, TIM-3) were coated on 96-well ELISA plates (Corning, #3690) overnight with 100 ng/well in PBS at 4°C, and blocked with 100 µl per well of 3% MPBS (PBS with 3% milk) at 37°C for 1 h. The plates were washed with PBS with 0.05% Tween 20 (PBST), then threefold serial diluted proteins were added and incubated at 37°C for 1.5 h. Plates were washed five times with PBST and 50 µl of 1:1,000 HRP conjugated anti-FLAG antibody (Sigma-Aldrich) in PBS were added per well before incubation at 37°C for 45 min. After extensive washes with PBST, the binding was measured by the addition of diammonium 2,2′-azino-bis (3-ethylbenzothiazoline-6-sulfonate) (ABTS) substrate (Roche Applied Science) and signal reading was carried out at 405 nm.

### FcRn Binding Measured by Bio-Layer Interferometry (BLI)

The binding of proteins to soluble human FcRn was measured by BLI using an Octet-Red96 system (Pall ForteBio) in 96-well plates (Greiner) at 37°C. Purified human FcRn (biotin labeled) was diluted in PBS with 0.02% Tween 20 (pH 7.4) and loaded onto streptavidin coated Dip-and-Read biosensor tips (Pall ForteBio) until saturation. Next, the tips were washed in the same buffer (60 s) for blocking unoccupied streptavidin-binding sites and were placed for 5 min in wells containing proteins. The proteins were diluted in PBS with 0.02% Tween 20 for the detection of binding at pH 7.4 or 6.0. A twofold dilution of proteins (mFc, 1-B10, 1-D1) was prepared from 10 µM to 0.625 µM. Then the sensor surfaces were regenerated with glycine buffer (pH 1.7) after 5 min of dissociation. The experiments included the following steps at 37°C: (1) equilibration step with assay buffer (PBS with 0.02% Tween 20, pH 7.4) for 10 min, (2) baseline step with assay buffer for 60 s, (3) loading step with 30 µg/ml biotin-FcRn in assay buffer for 5 min, (4) baseline step with assay buffer (pH 6.0/7.4) for 5 min, (5) association step with varying concentrations of proteins in assay buffer (pH 6.0/7.4) for 5 min, (6) dissociation step with assay buffer for 5 min, and (7) regeneration step with glycine buffer (pH 1.7) for 5 s, repeat three times. The curves were fitted based on the 1:1 binding kinetic model using ForteBio Data Analysis software.

### Statistics

Statistical analysis was performed using SPSS 19.0 software. The comparison of binding capability of the selected Fc mutants to various unrelated antigens were carried out by Student’s *t*-test. All comparisons were two-tailed and *p* < 0.05 was considered statistically significant.

## Results

### Construction of a Large Fc Mutant Library and Selection of Monomeric Fc

We have previously reported the generation of three mFc proteins using a combination of rational design and multiple panning/screening procedure (20, 21). Previous studies have focused on reducing the molecular weight of Fc. The resulting Fc monomers were found to possess high stability, FcRn-binding capability and similar serum half-life as compared to the wild-type IgG1 Fc *in vivo* (20). Despite this, we recently found that they displayed relatively strong non-specific binding to unrelated antigens at high protein concentration. As shown in Figure 1C, at a concentration of 2 µM, mFc evidently bound to all eight tested viral proteins and cancer-related antigens, including HIV-1 gp140, Zika virus (ZIKV) envelope protein domain II (EDII), domain III (EDIII), mesothelin, 5T4, PD-L1, OX40, TIM-3. We hypothesized the non-specificity came from the introduced mutations located at the original CH3 dimmerization interface of IgG1 Fc, and could be resolved by the change of mutation sites of mFc. Therefore, based on our knowledge of Fc homo-dimerization, we designed a phage display library that enables a combination of rational and random scanning mutagenesis of 7 IgG1 Fc residues (positions Leu-351, Thr-366, Leu-368, Pro-395, Lys-409, Met-428, and Asn-434). Among them, five residues (positions Leu-351, Thr-366, Leu-368, Pro-395, Lys-409) were previously found to greatly impact Fc dimerization, since some specific combinations of their mutations were able to result in soluble monomeric Fcs. In light of the experience gained during the development of monomeric Fcs (20, 21), we simultaneously mutated Leu-351 to Ser or unchanged, Thr-366 to Arg or His, Leu-368 to His or unchanged, Pro-395 to Lys or Arg, and Lys-409 to any of the 20 amino acids (random mutation). Meanwhile, two other residues (Met-428, Asn-434), the mutation of which has been reported to enhance FcRn binding, were randomly mutated to compromise the potential decrease of FcRn-binding activity due to the mutagenesis. Combination of these mutations resulted in a large phage display library containing 1.28 × 10^5^ unique IgG1 Fc mutants. The diversity was verified by randomly picked and sequenced 30 clones from this library (Figure 1D).

**Figure 2:**
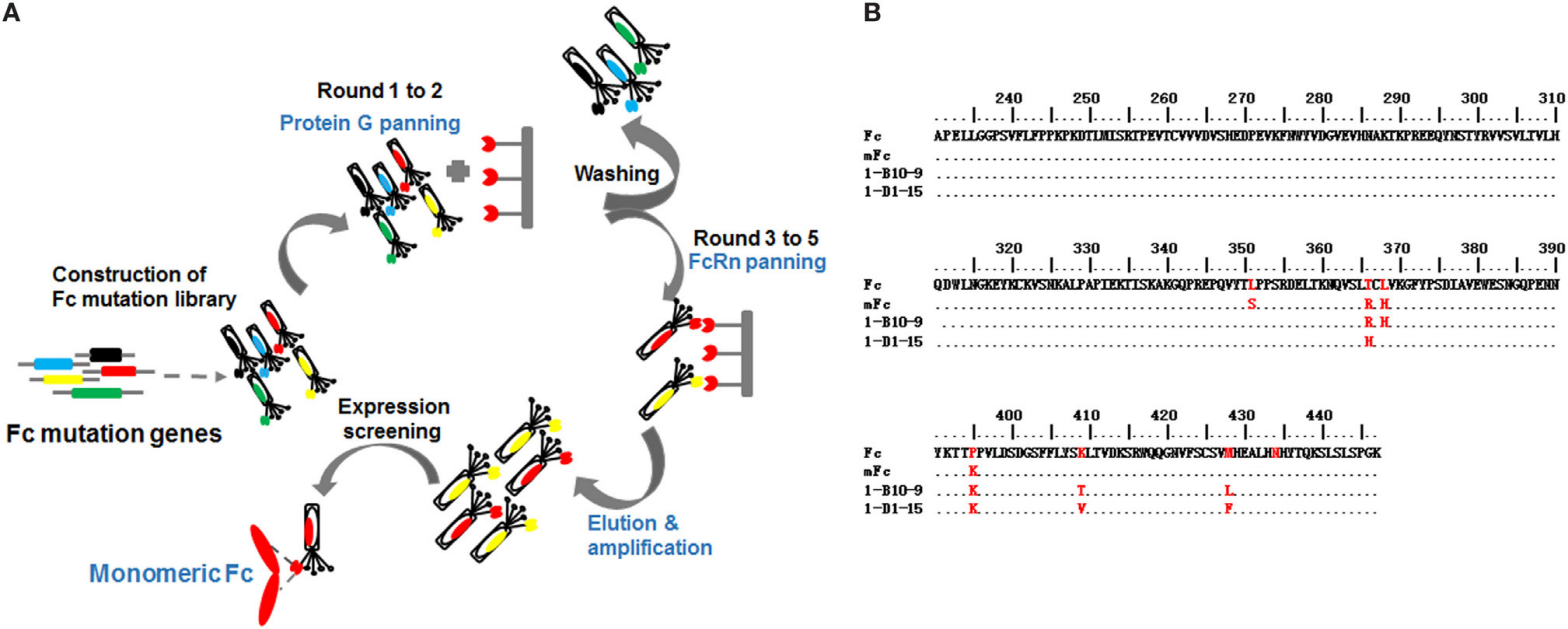
The schematic bio-panning process **(A)** and amino acid sequence alignment of screened IgG1 Fc mutants **(B)**.

In addition to the library design, we also improved the library panning procedures to generate clones with decreased non-specific binding (Figure 2A). In the previous studies, we screened for phage-displayed soluble and well-folded Fc mutants by purifying phages with protein G resin. The aggregated or non-structured mutants were not able to bind protein G and, therefore, were removed from the library. Although this screening was quite potent and effective, we realized that the mutants with high non-specificity can still bind protein G and thus retained in the following FcRn panning rounds. To address this issue, in the current study, instead of protein G resin we used protein G covalently attached to magnetic beads, which enables the harsh and thorough washing of the bound phages in solution. Moreover, we panned against protein G magnetic beads for two rounds, instead of one round of screening in the previous studies (20), enabling the enrichment of strong and specific protein G binders and, importantly, the removal of Fc mutants with non-specific bindings.

After two rounds of protein G panning, the enriched library was further panned against human FcRn for three additional rounds (Figure 2A). To isolate binders with pH-dependent FcRn binding, phages were washed using buffer at acidic pH (6.0) and eluted from beads using buffer at neutral pH (7.4). Using this procedure, 45 clones were selected from the final enriched library by monoclonal phage ELISA. Interestingly, the sequences of the Fc mutants were very diverse, suggesting that soluble Fc mutants with potent FcRn binding could be achieved by multiple combinational mutations.

**Figure 3:**
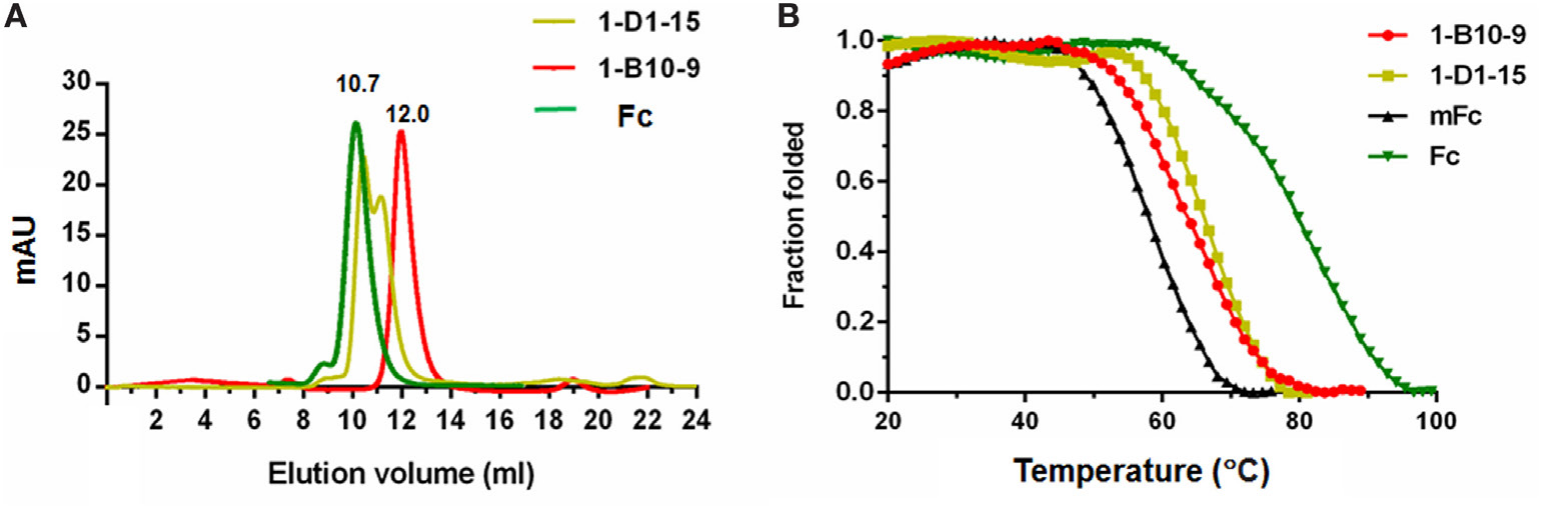
Size exclusion chromatography of Fc, 1-B10-9, 1-D1-15 **(A)**. Protein samples were loaded onto an analytical Superdex 75 10/300 GL column connected to an FPLC ÄKTA BASIC pH/C system (GE Healthcare) and plots of the change in fraction folded (calculated from circular dichroism molar ellipticity at 216 nm) for Fc, mFc, 1-B10-9, 1-D1-15 **(B)**.

Next, we assessed the oligomeric state of the Fc mutants by SEC. All these Fc mutants were highly expressed in soluble form in *E. coli*, and 10–20 mg purified protein could be obtained from 1 l bacterial culture under optional conditions. One Fc mutant, designated as 1-B10-9, was found to be pure monomeric, with a single monomer peak eluted at 12.0 ml (Figures 2B and 3A). Our previously reported monomeric Fc (molecular masses of 27 kDa) displayed an identical pattern with a single peak also at 12.0 ml (data not shown). All other mutants were found to exist as a mixture of monomer and dimer, as exemplified by 1-D1-15 (Figures 2B and 3A). Surprisingly, 1-B10-9 has Arg, His, and Lys at positions 366, 368, and 395, respectively, which are identical to the previously reported mFc. This result suggests that these mutants are essential to maintain a pure monomeric state. Different from mFc, 1-B10-9 has no mutation at position 351 as compared to the wild-type Fc, but has two additional mutations, K409T and M428L as compared to mFc (Figure 2B).

### Stability of Monomeric Fc

To detect the thermal stability of the Fc mutants, their CD ellipticity at 216 nm was measured as a function of temperature. The previously reported mFc and the wild-type dimeric IgG1 Fc were used as controls. Interestingly, we found that 1-B10-9 and 1-D1-15 were more stable than the previously identified mFc. The midpoint transition (melting) temperature (Tm) for mFc was 58.4 ± 0.2°C, which is in consistent with the previous report (54.3 ± 0.1°C) (21). The Tm for 1-B10-9, 1-D1-15, and Fc were 64.0 ± 0.1, 66.6 ± 0.2, and 80.6 ± 0.3°C, respectively (Figure 3B). These results suggest that different mutation sites affect the stability of IgG1 Fc mutants, and that the additional mutations identified on 1-B10-9 and 1-D1-15 may stabilize the constructs.

### The New Monomeric Fc Binds to Human FcRn

**Figure 4:**
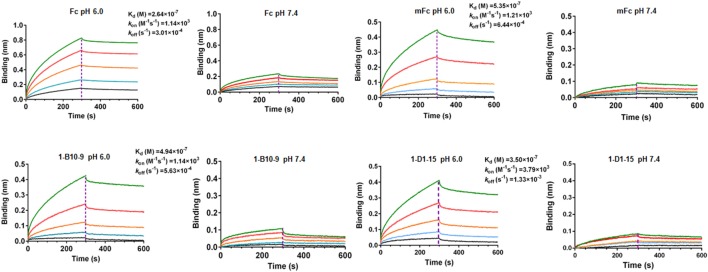
Binding affinity of Fc, mFc, 1-B10-9, and 1-D1-15 to neonatal Fc receptor (FcRn) was measured by Octet-RED (Pall ForteBio). Purified biotin-labeled human FcRn was immobilized on the streptavidin coated biosensors. The analytes consisted of serial dilution of proteins between 10 and 0.625 µM. Binding kinetics was evaluated using 1:1 Langmuir binding model by Fortebio Data Analysis 8.0 Software.

We next examined whether 1-B10-9 could bind to human FcRn in a pH-dependent manner as the wild-type Fc or mFc using BLI by Octet-RED (Pall ForteBio). Streptavidin coated biosensors immobilized with biotinylated human FcRn were exposed to different concentrations of 1-B10-9, 1-D1-15, mFc, and the wild-type Fc. The assay was carried out under pH 6.0 and pH 7.4, respectively, and the biosensors were regenerated with pH 1.7 buffer. As shown in Figure 4, the wild-type Fc and Fc mutants (mFc, 1-B10-9, 1-D1-15) displayed potent binding to FcRn at pH 6.0. The equilibrium dissociation constant (*K*_D_) of the wild-type Fc for FcRn was 0.26 µM with on-rate (*k*_on_) of 1.1 × 10^3^ M^−1^s^−1^ and off-rate (*k*_off_) of 3.0 × 10^−4^ s^−1^, which is comparable to that in the previous report (*K*_D_ of 0.11 µM) (20). The 1-B10-9 displayed very similar binding kinetics (*k*_on_ of 1.1 × 10^3^ M^−1^s^−1^, *k*_off_ of 5.6 × 10^−4^ s^−1^) to mFc (*k*_on_ of 1.2 × 10^3^ M^−1^s^−1^, *k*_off_ of 6.4 × 10^−4^ s^−1^), while the 1-D1-15 displayed slightly faster on-rate (3.8 × 10^3^ M^−1^s^−1^) and faster off-rate (1.3 × 10^−4^ s^−1^). All these three Fc mutants (mFc, 1-B10-9, 1-D1-15) exhibited very similar binding affinities to FcRn (*K*_D_ of 0.54, 0.49, and 0.35 µM, respectively). On the contrary, the wild-type Fc and the Fc mutants showed drastically reduced binding to FcRn when the assay was performed at pH 7.4 (Figure 4). Taken together, these results demonstrated that 1-B10-9 maintained the characteristic pH-dependent FcRn binding of IgG1 Fc.

### The New Monomeric Fc Has Significantly Decreased Non-Specific Binding

**Figure 5:**
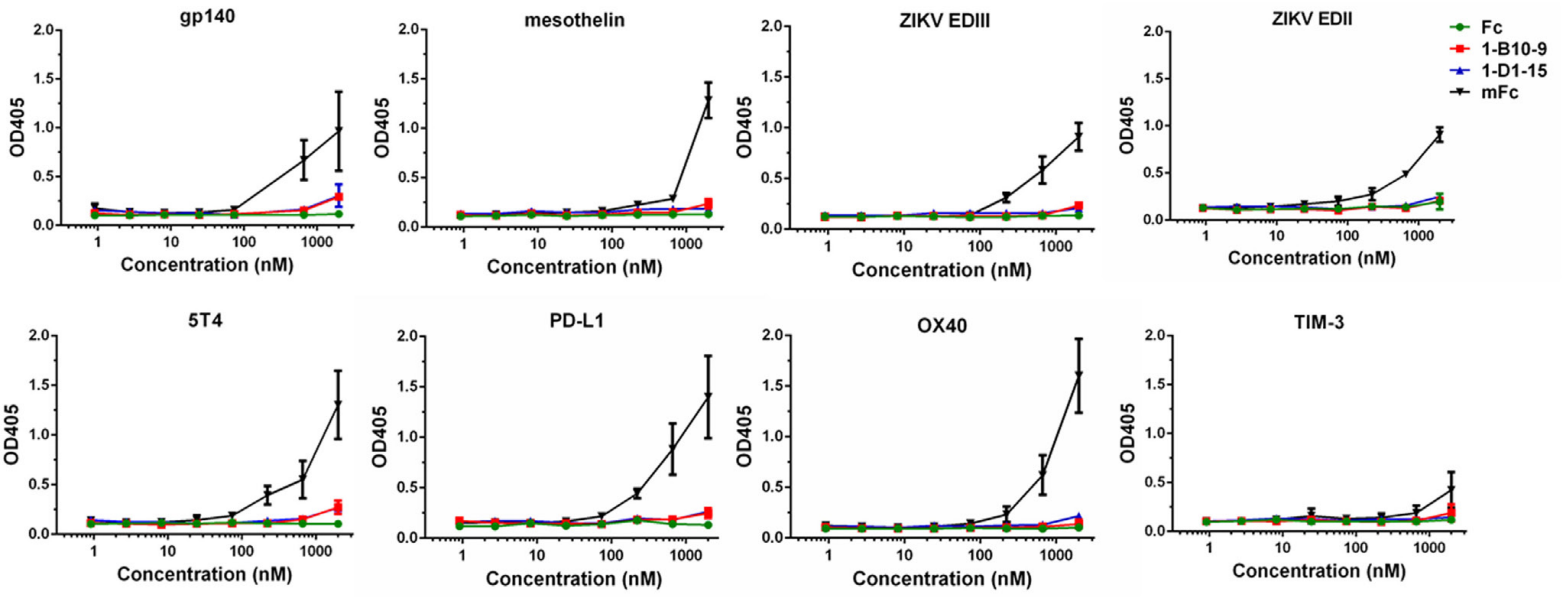
The binding of IgG1 Fc and Fc mutants (mFc, 1-B10-9, 1-D1-15) to recombinant viral proteins and cancer-related antigens (HIV-1 gp140, ZIKV EDII, ZIKV EDIII, mesothelin, 5T4, PD-L1, OX40, TIM-3) was measured by ELISA. The recombinant antigens were coated on ELISA plates, and HRP-conjugated anti-FLAG antibody was used for detection of Fc and Fc mutants. The error bars reflect the SD.

To evaluate the effectiveness of our strategy in reducing non-specific binding, we randomly selected a panel of viral or cancer-related antigens available in our laboratory, and measured the binding of IgG1 Fc and Fc mutants to these recombinant proteins. Such antigens include HIV-1 gp140, ZIKV glycoprotein EDII, EDIII, mesothelin, 5T4, PD-L1, OX40, and TIM-3. The ELISA plates were coated with these antigens and incubated with serial dilutions of Fc or Fc mutants. As show in Figure 5, the previously developed mFc displayed relatively strong non-specific binding to all these proteins especially at the highest concentration tested (2 µM). Strikingly, 1-B10-9, as well as 1-D1-15 and IgG1 Fc, showed significantly lower (*p* < 0.05) binding to these antigens at high concentrations (Figure 5). These results confirmed that the Fc mutants with significantly decreased non-specificity could be successfully identified using the present library-based panning approach.

## Discussion

In previous studies, we have described the generation of several monomeric Fcs (mFc.1, mFc.23, mFc.67, and mFc) by mutating four to seven critical residues located on the IgG1 Fc dimerization interface (20, 21). The monomeric Fcs bound FcRn comparably with the wild-type dimeric Fc, providing direct evidence that Fc dimerization is not required for effective binding to FcRn. This property is critical for the developability of monomeric Fc-based therapeutics, as various studies have demonstrated that half-life of an IgG depends on its pH-dependent binding to FcRn, and that Fc engineering to enhance FcRn binding is effective for elongating half-life or increasing cellular uptake of IgG (6, 14, 23, 24). Based on this finding, we have furthered our studies and developed a number of mFc-based or mFc-derived constructs (monomeric CH3, CH2-CH3 hybrids, etc.) and used them as fusion partners to generate monomeric fusion proteins (25–27). Some of these fusion proteins showed the improved transport across FcRn-expressing cells and did not induce Fc-mediated cytotoxicity *in vitro* (ADCC and CDC) (21). Meanwhile, we introduced point mutations in the CH2 domain and grafted CDR3 regions onto the CH3 domain of mFc, we successfully identified panels of high-affinity binders by panning mFc-based libraries against viral and cancer antigens (unpublished data). The size of mFc is largely reduced compared with the wild-type Fc, and therefore, the mFc-based fusion proteins or antigen binders could achieve enhanced tissue penetration and wider range of possible targets, as well as lower production costs.

In spite of these advantages, in the follow-up study, we found that the binding to unrelated proteins was generally observed for such monomeric Fcs, raising potential risks for their clinical application due to the immunogenicity and pharmacokinetics-related issues. For instance, the unwanted formation of antigen–antibody immune complexes can elicit a variety of downstream effects and further immunogenic responses (28). Their levels, kinetics of interaction, size, polyclonal diversity, distribution, elimination, and antibody-mediated physiological effects can be potentially translated to clinically observable adverse effects (29, 30). Besides, unrelated proteins could bind to the FcRn-binding site of the monomeric Fcs, thereby inhibiting FcRn-mediated recycling of fusion proteins or mFc-based binders. We reasoned that the non-specificity came from the introduced mutations during the generation of monomeric Fc. Therefore, in the present study, we re-designed a phage-displayed Fc mutant library that enables a combination of rational and random scanning mutagenesis of seven mFc residues, and largely improved our panning and screening procedure with a focus to remove non-specific binding. Using these strategies, we identified a new monomeric Fc, 1-B10-9, that displayed significantly decreased non-specificity. In addition, it exhibited higher thermal stability than mFc, and comparable pH-dependent FcRn-binding capability.

Several conclusions can be drawn from these results. First, the successful generation of new monomeric Fc with minimal non-specificity demonstrates the robust performance of the present approach in reducing non-specific protein binding. One major difference to the previous strategies probably comes from the introduction of Protein G magnetic beads to enrich for the strongest Protein G binders. In contrast to the previous studies using Protein G resin only to remove unstructured mutants (20), the application of Protein G beads enabled harsh and adjustable washing conditions to remove non-specific binders and to result in focused enrichment of Fc mutants with the most potent Protein G binding. Only neglectable binding to unrelated antigens was detected not only for the dimeric Fc and the monomeric 1-B10-9 but also for 1-D1-15, the Fc mutant existing as a mixture of monomer and oligomer as suggested by SEC analysis (Figures 3A,5). This result suggests that the non-specificity was from the introduced mutation amino acids, rather than the monomeric nature of Fc mutants. Interestingly, we found that the non-specificity was probably not due to the hydrophobicity of these residues, as all the Fc mutants possess comparable calculated hydrophobicity index (mFc, −0.654; 1-B10-9, −0.610; 1-D1-15, −0.553). Second, the current display-based strategy allows for the examination of each mutation residues related to the functionality of IgG1 Fc mutants. This can be achieved by rationally or randomly mutating the residue of interest in a displayed mutation library and subsequently analyzing the sequences enriched from bio-panning for those with particular properties. For instance, whereas the non-monomeric Fc mutants have diverse sequences, the identified new IgG1 monomeric Fc, 1-B10-9, has the identical T366R and L368H mutations to the previous reported mFc, suggesting the essential roles of these two mutations in maintaining monomeric status. It is possible that a positively charged surface formed by these two mutations plays a role in the disruption of dimerization. The 1-D1-15, in contrast, has a histidine at residue 366 but a leucine at residue 368, and exists in a non-monomeric form. Besides, the other two residues that located at the CH3 dimerization interface (residues 351 and 409) may play a crucial role in the non-specific binding of Fc mutants. Of note, only the previous-developed mFc has a L351S mutation, suggesting that the Ser-351 residue *per se* or the local conformational change introduced by this mFc mutation may contribute to its relative strong non-specific binding to unrelated targets.

In summary, this study suggests that the new monomeric IgG1 Fc, 1-B10-9, could be potentially applicable in the engineering and development of novel biopharmaceuticals due to its high stability, pH-dependent FcRn binding, and significantly decreased non-specificity compared with the previous reported monomeric Fc mutants.

## Author Contributions

TY and CW conceived and designed the project. CW, YW, LW, YJ, and GC carried out the experiments. CW, BH, DH, YK, AH, and GH analyzed the data. TY and CW wrote the paper with input from all co-authors.

## Conflict of Interest Statement

The authors declare that the research was conducted in the absence of any commercial or financial relationships that could be construed as a potential conflict of interest.
